# NGF Is an Essential Survival Factor for Bronchial Epithelial Cells during Respiratory Syncytial Virus Infection

**DOI:** 10.1371/journal.pone.0006444

**Published:** 2009-07-31

**Authors:** Sreekumar Othumpangat, Laura F. Gibson, Lennie Samsell, Giovanni Piedimonte

**Affiliations:** 1 Department of Pediatrics and Pediatric Research Institute, West Virginia University School of Medicine, Morgantown, West Virginia, United States of America; 2 Mary Babb Randolph Cancer Center, West Virginia University School of Medicine, Morgantown, West Virginia, United States of America; 3 Department of Microbiology, Immunology, and Cell Biology, West Virginia University School of Medicine, Morgantown, West Virginia, United States of America; Abramson Research Center, United States of America

## Abstract

**Background:**

Overall expression of neurotrophins in the respiratory tract is upregulated in infants infected by the respiratory syncytial virus (RSV), but it is unclear where (structural vs. inflammatory cells, upper vs. lower airways) and why, these changes occur. We analyzed systematically the expression of neurotrophic factors and receptors following RSV infection of human nasal, tracheal, and bronchial epithelial cells, and tested the hypothesis that neurotrophins work as innate survival factors for infected respiratory epithelia.

**Methodology:**

Expression of neurotrophic factors (nerve growth factor, NGF; brain-derived neurotrophic factor, BDNF) and receptors (trkA, trkB, p75) was analyzed at the protein level by immunofluorescence and flow cytometry and at the mRNA level by real-time PCR. Targeted siRNA was utilized to blunt NGF expression, and its effect on virus-induced apoptosis/necrosis was evaluated by flow cytometry following annexin V/7-AAD staining.

**Principal Findings:**

RSV infection was more efficient in cells from more distal (bronchial) vs. more proximal origin. In bronchial cells, RSV infection induced transcript and protein overexpression of NGF and its high-affinity receptor trkA, with concomitant downregulation of the low-affinity p75^NTR^. In contrast, tracheal cells exhibited an increase in BDNF, trkA and trkB, and nasal cells increased only trkA. RSV-infected bronchial cells transfected with NGF-specific siRNA exhibited decreased trkA and increased p75^NTR^ expression. Furthermore, the survival of bronchial epithelial cells was dramatically decreased when their endogenous NGF supply was depleted prior to RSV infection.

**Conclusions/Significance:**

RSV infection of the distal airway epithelium, but not of the more proximal sections, results in overexpression of NGF and its trkA receptor, while the other p75^NTR^ receptor is markedly downregulated. This pattern of neurotrophin expression confers protection against virus-induced apoptosis, and its inhibition amplifies programmed cell death in the infected bronchial epithelium. Thus, pharmacologic modulation of NGF expression may offer a promising new approach for management of common respiratory infections.

## Introduction

The most common agent of lower respiratory infections in early childhood is respiratory syncytial virus (RSV) [1], [2], [3]. In addition to the significant morbidity and mortality caused by the acute infection [4], a large proportion of these young patients continue to have recurrent post-bronchiolitis episodes of lower airway obstruction, which may continue for years after the acute infection has resolved [4], [5], [6]. More recently, RSV has been shown to be a significant cause of respiratory illness among elderly and high-risk adults [7], and studies in patients with chronic obstructive pulmonary disease (COPD) have raised the possibility of persistent low-grade RSV infection in this population [8].

Studies conducted in animal models have shown that RSV infection increases the expression of critical neurotrophic growth factors and receptors in the lungs, and that the consequent changes in airway neuro-immunomodulation play an important role in the pathophysiology of airway inflammation and hyperreactivity during and after the acute infection [9]. We have recently confirmed these findings in humans, showing increased concentrations of nerve growth factor (NGF), brain-derived neurotrophic factor (BDNF), and the receptor tropomyosin-related kinase A (trkA) in the cell fractions obtained by bronchoalveolar lavage from infants with RSV infection [10]. However, we were unable to define whether the source of these factors was in infected proximal or distal structural cells, or inflammatory cells recruited and stimulated by the virus. Also, the exact role played by neurotrophins in inflammatory conditions has not been established conclusively, and there is circumstantial evidence supporting both protective and pathologic effects [2], [11], [12].

Although multiple cell types are involved in the pathogenesis of RSV disease [13], the respiratory epithelium is unanimously believed to be the primary target of the infection and plays a central role in airway injury and remodeling by releasing inflammatory and growth factors capable of modulating immune and reparative processes [14], [15], [16]. It is common knowledge that naturally occurring RSV infections in humans initiate in the nasal region, and in young children frequently spread through the trachea and finally establish in the bronchial tree. The individual cellular responses to RSV may differ both quantitatively and qualitatively among different anatomical regions of the respiratory system, but specific information is difficult to obtain because most clinical data are derived from lavage samples collected from the upper [17] or lower airways [10] of RSV-infected patients, therefore pooling together information from different sections of the respiratory tract and bypassing others.

Thus, in this study we evaluated human cell lines deriving from nasal, tracheal or bronchial epithelium, studied their individual susceptibility to RSV infection, and systematically quantified the effects of the infection on the transcript and protein expression of different neurotrophic factors and receptors by immunocytochemistry, flow cytometry, and real-time PCR. Extending these studies, we also explored the functional consequences of selective NGF knock-out by siRNA in infected respiratory epithelia, and specifically tested the hypothesis that this neurotrophin contributes to an innate survival strategy deployed by cells being infected against the invading virus, thereby modulating the pathological and clinical manifestations of the infection.

## Methods

### Virus

RSV derived from RSV-A_2_ expressing the gene for green fluorescent protein (rgRSV) was a kind gift of Dr. Mark E. Peeples (Columbus Children Research Institute, Columbus, OH) and Dr. Peter Collins (National Institutes of Health, Bethesda, MD). Cultivation and harvesting of rgRSV was performed as described previously. Briefly, virus was serially diluted and added to HEp-2 cell monolayers in 96 well-plates. Cells were incubated for 22 h and green cells were counted by fluorescent microscopy. RSV titer was determined in a modified plaque-forming unit (PFU) assay, and was calculated as follows: (number of infectious units)×(inverse of dilution)/(volume of inoculum). The stock titer of the virus pool used in the current study was 4.7×10^6^ PFU/ml. To confirm that the observed effects were caused by actively replicating virus and not by exposure to constituents of the virion or culture medium, selected experiments were performed using aliquots of rgRSV irradiated with a UV light source for 20 min to inactivate the viral nucleic acid [18].

### Cell lines

Human nasal, tracheal, and bronchial epithelial cells from multiple donors were purchased from PromoCell (Heidelberg, Germany) and were maintained in the recommended media and supplements provided by the vendor. Each experiment was repeated using cells from different donors throughout the study to control for host genetics and environment. The number of passage times for each airway section was matched in each experiment and it never exceeded 5 passages. As 100% confluency alters the cells morphology, we performed our experiments in cells that were 70–80% confluent at the time of the infection with rgRSV at MOI (multiplicity of infection) ranging from 1 to 10. Virus multiplication was periodically monitored using a florescent microscope.

### FACS analysis

Nasal, tracheal and bronchial epithelial cells were grown to confluency in a 6-well plate and were infected with rgRSV at 1 MOI. After 48 h of infection, cells were isolated by trypsinization and were stained for intracellular and surface detection of NGF, BDNF and their receptors trkA, trkB, and p75^NTR^. TrkB and p75^NTR^ antibodies were purchased from Santa Cruz Biotechnology (Santa Cruz, CA). TrkA and isotype control goat IgG conjugated with allophycocyanin (APC) were obtained from R&D Systems (Minneapolis, MN). The additional isotype control rabbit IgG was purchased from Southern Biotechnology (Birmingham, AL). We used antibodies conjugated with APC or phycoerythrin (PE) because the virus was labeled with green fluorescent protein (GFP). For cell death analysis, gating was adjusted using cyanine 3 (Cy3) or 7-aminoactinomycin D (7-AAD) staining with dot plots displaying FL3-7-AAD on the y-axis and FL2-Cy3 on the x-axis, and 10,000 events were collected for each sample. The five parameters simultaneously collected were linear forward-angle light scatter (FSC), linear side scatter (SSC), log FITC, log Cy3, and log 7-AAD. Data were acquired using a FACSCalibur flow cytometer with Cell Quest Pro software (BD Biosciences, Franklin Lakes, NJ) and were analyzed with Windows Multiple Document Interface (WinMDI) software for flow cytometry version 2.9 (The Scripps Research Institute, La Jolla, CA).

### Immunofluorescence

Nasal, tracheal and bronchial epithelial cells were grown to 70–80% confluence on glass coverslips in a 6-well plate and infected with 1 MOI of rgRSV for 48 h. Following treatment, cells were rinsed in phosphate-buffered saline (PBS) and fixed in 4% formaldehyde at room temperature for 10 minutes. Permeabilization of cells was obtained with 0.5% Triton X-100 for 20 minutes. Nonspecific antibody binding was blocked by incubation for 20 minutes in 1× PBS/5% BSA. The coverslips were stained for 1 h with anti-human NGF antibody (Santa Cruz Biotechnology) in PBS/5% BSA or with the corresponding control isotype, followed by Alexa Fluor 555 donkey anti-rabbit antibody (Invitrogen, Carlsbad, CA) for another hour. The nuclei were stained with TOPRO-3 (Invitrogen). Coverslips were inverted on slides and mounted with Prolong Gold anti-fade reagent (Invitrogen). Images were obtained using a Zeiss LSM510 confocal microscope with an AxioImager Z1 system (Carl Zeiss, Jena, Germany). The FITC (488 nm) channel was used to observe the GFP protein expressed by rgRSV-infected cells.

### Real-time PCR analysis

Total RNA was isolated using an RNeasy kit following the manufacturer's recommendations (Qiagen Inc., Valencia, CA). Total RNA (1 µg) was used as template for cDNA synthesis (Applied Biosystems, Foster City, CA) and the cDNAs were used for quantitative real-time PCR analysis using SYBR green master mix (Applied Biosystems) with an ABI 7500 real-time cycler (Applied Biosystems). NGF, BDNF, trkA, trkB and p75^NTR^ primers were purchased from SuperArray (Rockville, MD). RSV primers specific for an 82-bp fragment of the viral nucleocapsid protein (sense: GCTCTTAGCAAAGTCAAGTTGAATGA; anti-sense: TGCTCCGTTGGATGGTGTATT) were purchased from Invitrogen. Hypoxanthine phosphoribosyltransferase 1 (HPRT1) was used as housekeeping gene for transcripts normalization and amplified with primers purchased from RealTimePrimers (Elkins Park, PA). Relative changes in gene expression were calculated with the following formula: fold change = 2̂^−(ddCt)^ = 2-dCt (treated samples)−dCt (control samples), where dCt = Ct (detected gene)−Ct (HPRT1) and Ct is the threshold number.

### NGF knock-down by siRNA

The interactions between NGF and the other neurotrophic factors and receptors during rgRSV infection were investigated by silencing its expression in epithelial cells with a specific siRNA (Dharmacon, Chicago, IL). Exponentially growing cells were transiently transfected with either 50 µM of NGF siRNA or the scrambled siRNA control using Lipofectamine 2000™ (Invitrogen). Transfected cells were monitored by real-time PCR for interleukin 6 (IL-6) gene expression (SuperArray), which is commonly used to rule out innate inflammatory activity triggered by double-stranded RNA in mammalian systems [19]. Forty-eight hours following transfection, cells were infected with 1 MOI of rgRSV for an additional 24 h. Following virus challenge, cells were collected by trypsinization and the pellet was used for RNA extraction.

### Cell death analysis

To evaluate whether NGF plays a role in the survival of epithelial cells during RSV infection, bronchial cells were treated with NGF siRNA or with the control scrambled siRNA for 48–60 h and the efficiency of NGF knock-down was monitored by real-time PCR analysis. NGF-deficient bronchial epithelial cells were infected with 1 or 5 MOI of rgRSV. After 24 h infection, cells were harvested, washed twice with PBS and counted using the trypan blue method for viability. Subsequently, cells were re-suspended in 500 µl of 1× annexin buffer and stained with 1 µl of annexin V-PE (MBL, Woburn, MA) for 10 min. In separate experiments, cells were treated with both 7-AAD (Enzo Life Sciences, Farmingdale, NY), a fluorescent intercalating dye that allows DNA quantitation, and annexin V-Cy3 for 15 min. After fixation with 1% paraformaldehyde, 500 µl of 1× annexin buffer were added to each sample and fluorescence flow cytometric analyses were performed using a BD Biosciences FACSCaliber instrument.

### Statistical analysis

Data are expressed as mean±SEM. Geometric mean fluorescent intensity (MFI) was calculated from the flow cytometry measurements obtained in 3 or 4 independent experiments. PCR data are the average of 3 or 4 independent experiments performed in duplicate. Paired Student's *t* test [20] was used to analyze differences between rgRSV-infected or UV-RSV-exposed cells and virus-free controls. Differences between NGF-specific vs. scrambled siRNA and apoptosis vs. necrosis were analyzed by ANOVA [21] and post-hoc pairwise comparisons between means [22] were performed using the Holm-Sidak method or the Sheffe's test. Statistical analysis was performed using the software SigmaStat version 3.5 (Systat Software, Point Richmond, CA) and StatView version 5.0.1 (SAS Institute, Cary, NC, USA) for Windows. Differences having a p value<0.05 were considered significant.

## Results

**Figure 1:**
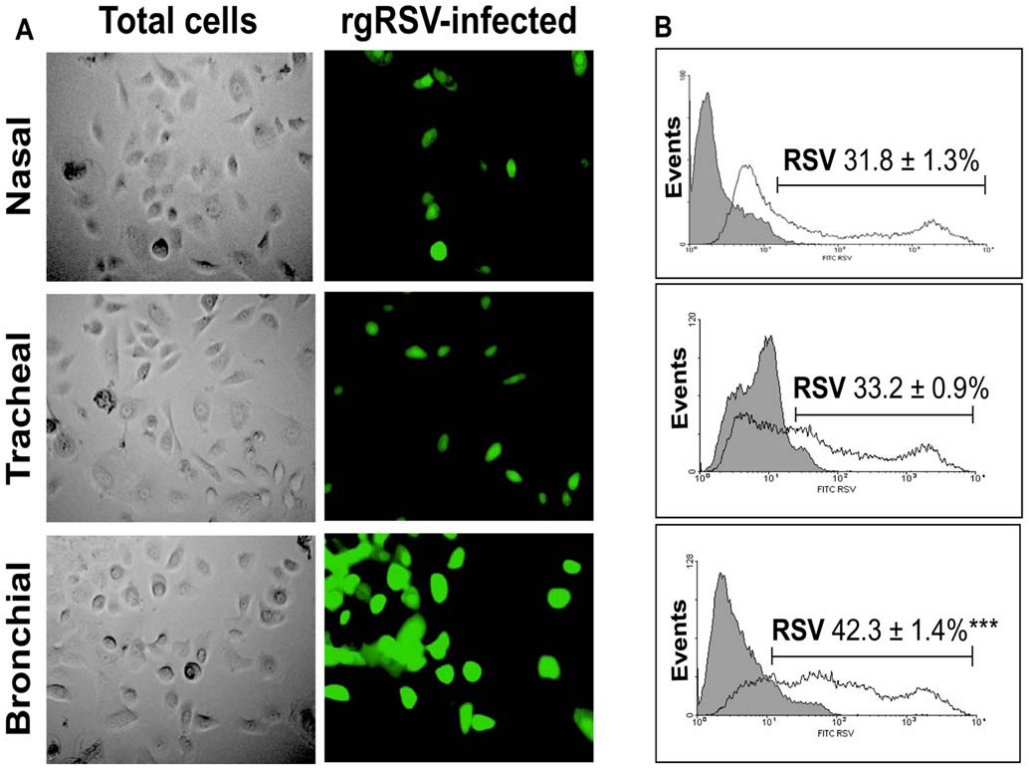
RSV infects bronchial epithelial cells more efficiently. (A) Human nasal, tracheal, and bronchial epithelial cells after infection with GFP-expressing RSV (rgRSV) at 1 MOI for 48 h. The bright field panels (left) show the total number of cells. The green fluorescent cells (center) represent those which are actively infected with RSV. (B) Bronchial epithelial cells are the most susceptible to RSV infection. Flow cytometric data show the percentage of fluorescent (infected) cells in each panel compared to the non-infected control cells (shaded histogram). Data are expressed as the mean±SEM (n = 4 experiments). *** = *p*<0.001 compared to nasal or tracheal cells.

We quantified the susceptibility of human-derived nasal, tracheal and bronchial epithelial cells to infection by recombinant human RSV expressing green fluorescent protein (rgRSV). As shown in Figure 1A, after incubation with rgRSV at 1 MOI for 48 h the efficiency of infection was significantly different among cell lines, with bronchial cells showing the highest infection rate compared to tracheal cells (p<0.001) and nasal cells (p<0.001). No significant difference was found between tracheal and nasal cells (p = 0.14).

**Figure 2:**
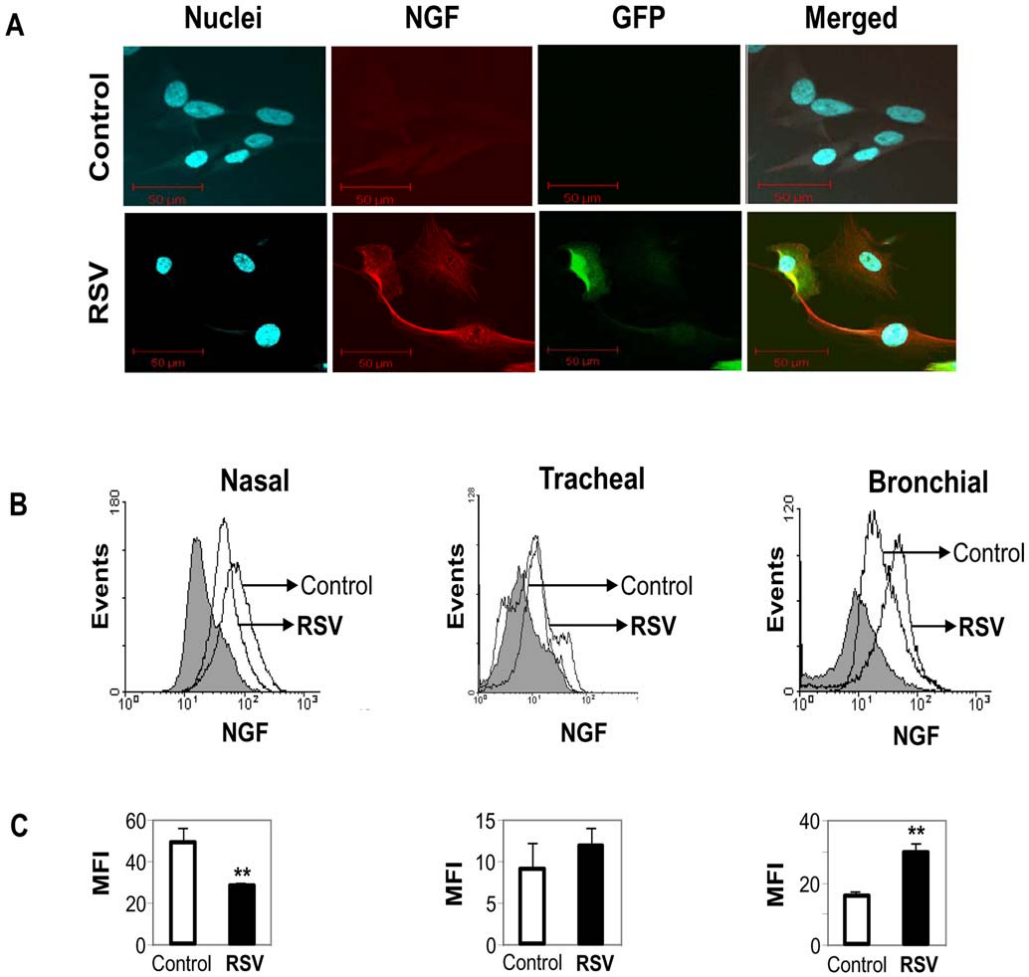
RSV infection modulates NGF expression. (A) RSV-induced NGF overexpression in human bronchial epithelial cells infected for 48 h. Cells were incubated with rabbit polyclonal NGF antibody for 1 h and subsequently treated with a secondary PE-conjugated anti-rabbit antibody. Nuclei were stained with TOPRO-3. Pictures were taken using a confocal microscope having a fluorescence emission of 488 nm for detection of green fluorescent protein (GFP). (B) Changes in NGF protein expression were detected by staining rgRSV-infected or non-infected nasal, tracheal and bronchial epithelial cells with anti-human NGF antibody or a matched isotype control (shaded histogram). Primary antibody binding was detected by incubation with PE-conjugated goat anti-rabbit antibody. (C) Geometric mean fluorescent intensity (MFI) is presented in bar graphs. Data are expressed as the mean±SEM (n = 4 experiments). ** = *p*<0.01 compared to non-infected controls.

Immunocytochemical analysis showed sharply increased NGF expression in rgRSV-infected bronchial epithelial cell cultures (Figure 2A), with non-infected cells having virtually undetectable NGF expression at baseline. Importantly, there was no visible change in immunoreactivity for NGF in rgRSV-infected nasal and tracheal cells compared to their non-infected controls (data not shown). Furthermore, NGF expression appeared strongly upregulated in infected bronchial cells with faint GFP staining, suggesting either a paracrine effect or NGF amplification even at low levels of viral replication. To quantitate the changes in protein expression, rgRSV-infected cells were stained for NGF and analyzed by FACS (Figure 2B and 2C). Again, bronchial epithelial cells infected with rgRSV expressed higher NGF protein levels than the non-infected control cells (p<0.01). Surprisingly, nasal epithelial cells infected by rgRSV showed a decrease in NGF expression (p<0.01), whereas tracheal cells showed no change compared to the non-infected control cells (p = 0.27).

**Figure 3:**
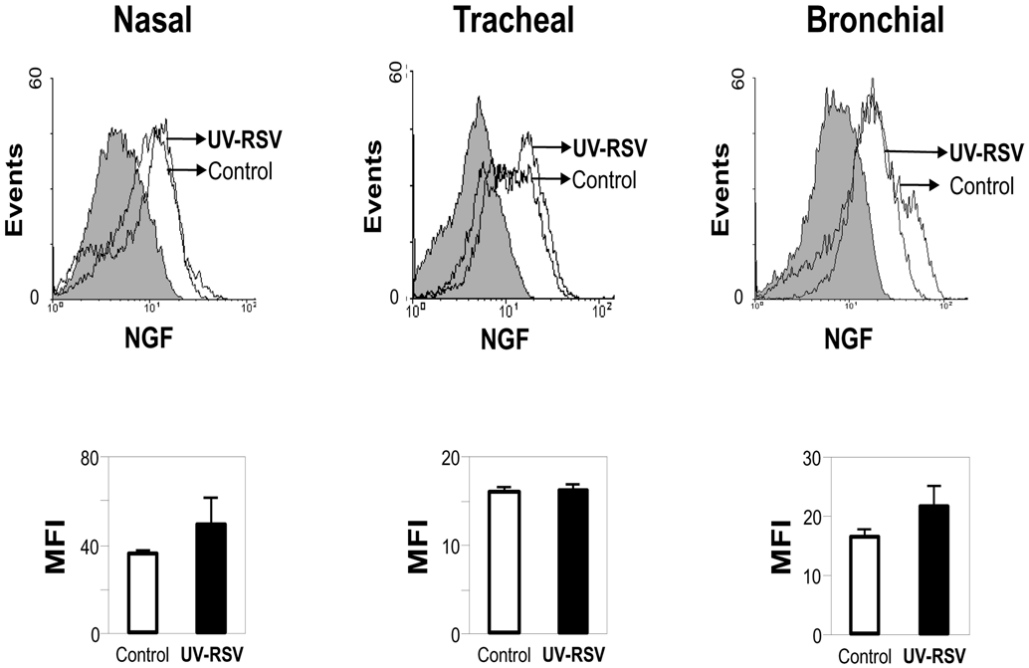
UV-inactivated RSV does not change NGF expression. (A) Following inactivation by UV light, rgRSV lost its effect on NGF expression in human airway epithelial cells. Changes in NGF protein expression were detected by staining UV-RSV-exposed or non-infected nasal, tracheal and bronchial epithelial cells with anti-human NGF antibody or a matched isotype control (shaded histogram). Primary antibody binding was detected by incubation with PE-conjugated goat anti-rabbit antibody. (B) Geometric mean fluorescent intensity (MFI) is presented in bar graphs. Data are expressed as the mean±SEM (n = 4 experiments). None of the effects was statistically significant.

Airway epithelial cells exposed to 1, 5, or 10 MOI of UV-inactivated rgRSV did not show any significant change in NGF expression compared to non-infected controls, indicating that neurotrophin upregulation requires active viral replication. Figure 3 shows the data obtained with the highest inoculum (10 MOI), which had no significant effect on NGF expression in nasal (p = 0.08), tracheal (p = 0.39), or bronchial (p = 0.11) cells.

**Figure 4:**
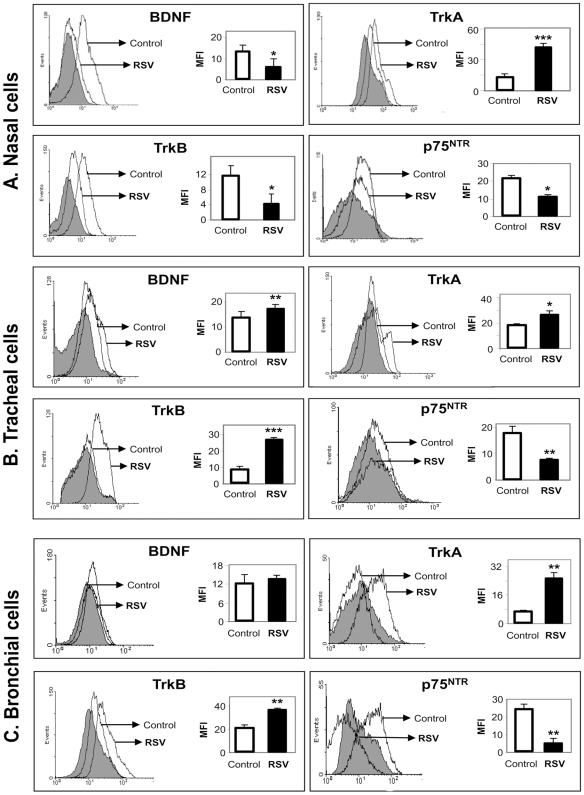
RSV-induced modulation of neurotrophic growth factors and their receptors in (A) nasal epithelial cells; (B) tracheal epithelial cells; (C) bronchial epithelial cells. Confluent plates of epithelial cells were infected with 1 MOI of rgRSV; after 6 h, the excess viral particles were removed and fresh culture medium added. Subsequently, cells were harvested at 48 h and stained with specific antibody or its matched isotype control (shaded histogram). Cells not exposed to rgRSV were used as controls. Changes in protein expression were measured by flow cytometry using suitable secondary antibodies conjugated with phycoerythrin (PE) or allophycocyanin (APC). Geometric mean fluorescent intensity (MFI) is presented in bar graphs. Data are expressed as the mean±SEM (n = 4 experiments). * = *p*<0.05; ** = *p*<0.01; *** = *p*<0.001 compared to non-infected controls.

To analyze the effect of RSV infection on the expression of the other neurotrophic factor BDNF, and of the neurotrophin receptors trkA, trkB, and p75^NTR^, we performed FACS analysis using specific antibodies. Data presented in Figure 4A show significant induction of the high-affinity NGF receptor trkA (p<0.001) in nasal epithelial cells infected by rgRSV, combined with downregulation of BDNF (p<0.05), its high-affinity receptor trkB (p<0.05), and the low-affinity pan-neurotrophin receptor p75^NTR^ (p<0.05). Tracheal epithelial cells (Figure 4B) exhibited an increased expression of trkA (p<0.05), BDNF (p<0.01) and trkB (p<0.001), but again with lower levels of p75^NTR^ (p<0.01). TrkA and trkB receptors were significantly upregulated (p<0.01) in rgRSV-infected bronchial cells (Figure 4C), but the p75^NTR^ receptor was significantly downregulated (p<0.01) in these cells as well. Overall, rgRSV consistently modified the pattern of NGF receptors expression in the airways, increasing the high-affinity trkA while decreasing the low-affinity p75^NTR^, whereas the effect on BDNF and its trkB receptor was generally smaller and more variable.

**Figure 5:**
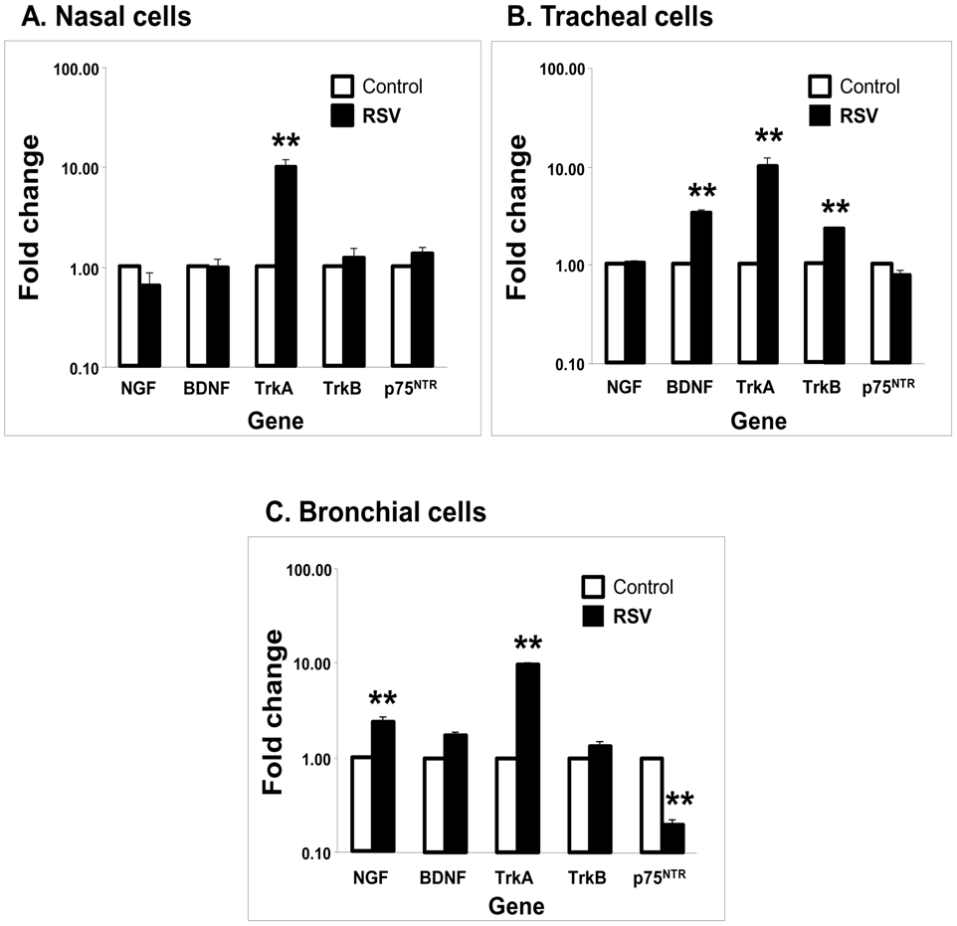
RSV-induced changes in neurotrophins gene expression in (A) nasal epithelial cells; (B) tracheal epithelial cells; and (C) bronchial epithelial cells. Cells were infected with 1 MOI of rgRSV for 24 h; then, RNA was isolated and cDNA synthesized for real-time PCR analysis. C_t_ values were normalized to the housekeeping gene HPRT1. Data shown are the average of 4 independent experiments performed in duplicate, and are presented as the mean±SEM (n = 8). ** = *p*<0.01 compared to non-infected controls.

Next, we analyzed whether protein expression was concordant with transcript levels in these cell lines following rgRSV infection. Real-time PCR analysis showed good correlation between mRNA and protein expression of NGF and trkA. Nasal cells (Figure 5A) infected with rgRSV did not exhibit any significant change in the transcripts of neurotrophic factors and receptors, with the exception of trkA that increased 8 fold over the non-infected controls (p<0.01). In tracheal cells (Figure 5B), both BDNF (p<0.01) and its receptor trkB (p<0.01) were significantly upregulated by rgRSV infection, along with the increase in trkA (p<0.01) and without change in p75^NTR^ levels. NGF was significantly upregulated (p<0.01) in rgRSV-infected bronchial cells (Figure 5C), together with a 9-fold increase in trkA (p<0.01). Also, bronchial cells uniquely showed significant downregulation of p75^NTR^ transcript (p<0.01) following rgRSV infection, consistent with the protein results.

**Figure 6:**
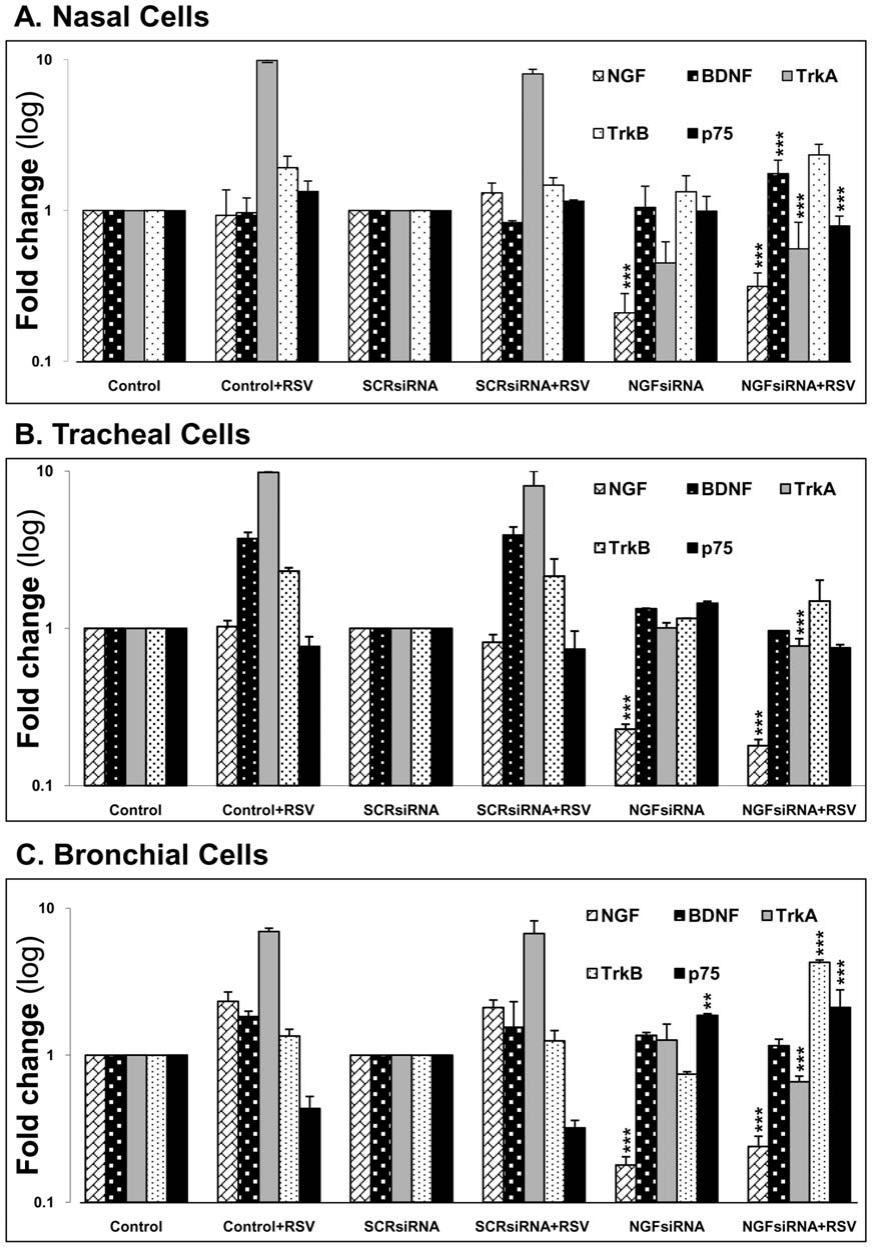
NGF knock-down alters gene expression of other neurotrophins in (A) nasal epithelial cells; (B) tracheal epithelial cells; and (C) bronchial epithelial cells. After 48 h transfection with either scrambled siRNA (SCRsiRNA) or NGF-specific siRNA (NGFsiRNA), cells were infected with 1 MOI of rgRSV for 24 h. Each experiment included non-transfected and non-infected controls. Transcript levels were analyzed by real-time PCR for relative changes in NGF, BDNF, trkA, trkB, and p75^NTR^ gene expression, and fold change was calculated by normalizing C_t_ values to HPRT1. Data shown are the average of 3 independent experiments performed in duplicate, and are presented as the mean±SEM (n = 6). ** = *p*<0.01; *** = *p*<0.001 compared to matched non-transfected controls.

To investigate the role of NGF in modulating the signaling of neurotrophic factors and their cognate receptors, non-infected or rgRSV-infected epithelial cells were transfected with scrambled control siRNA or NGF-specific siRNA. As shown in Figure 6, non-infected nasal, tracheal and bronchial cells transfected with scrambled siRNA did not show any change in neurotrophin gene expression compared to non-transfected non-infected cells. Similarly, transfection of rgRSV-infected cells with scrambled siRNA did not cause significant changes compared to non-transfected infected cells. Also, transfection of epithelial cells with either scrambled (p = 0.47) or NGF-specific siRNA (p = 0.16) did not induce IL-6 mRNA expression (data not shown). NGF knock-down by siRNA was efficient (70–90%) and highly statistically significant (p<0.001) in all three cell lines evaluated, and affected the expression of other neurotrophic factors and receptors during rgRSV infection. In particular, NGF depletion in bronchial cells reversed the effects of rgRSV on both its high-affinity receptor trkA (p<0.001) and low-affinity receptor p75^NTR^ (p<0.001).

**Figure 7:**
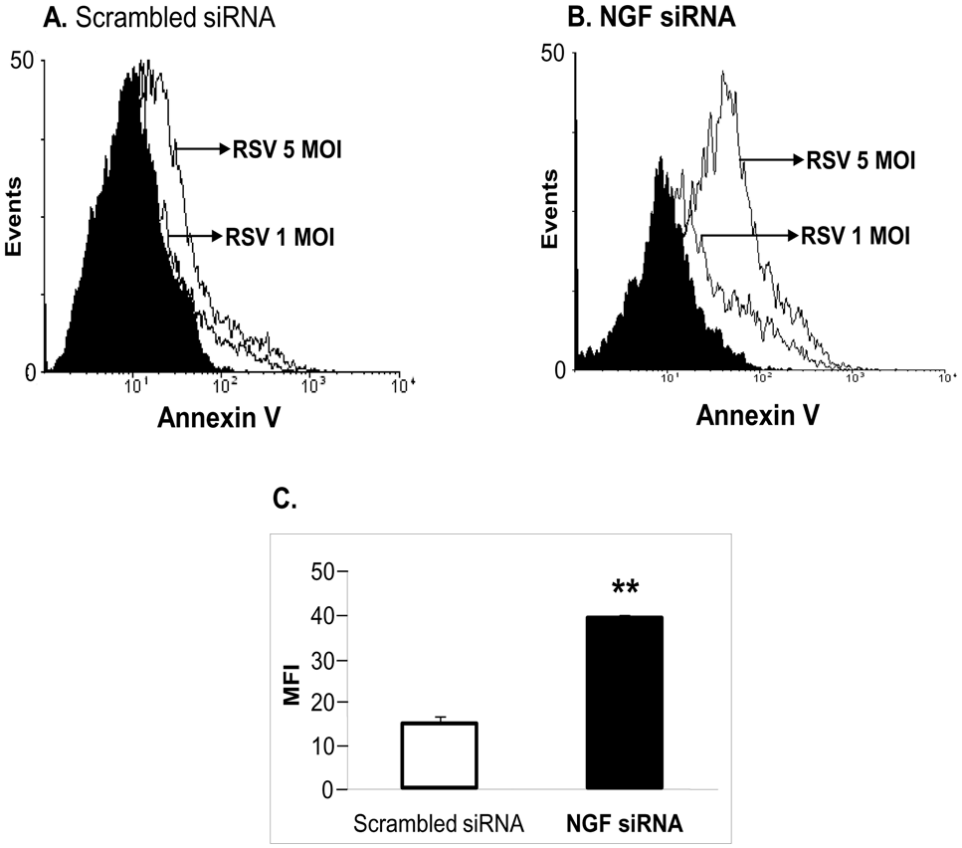
Apoptosis in RSV-infected bronchial epithelial cells increases after NGF knock-down. (A) Scrambled siRNA-transfected bronchial cells infected with 1 MOI or 5 MOI of rgRSV. (B) NGF siRNA-transfected bronchial cells infected with 1 MOI or 5 MOI of rgRSV. After 24 h of infection, cells were analyzed for apoptosis by flow cytometry with annexin V PE-conjugated antibody. The shaded portions of the histograms represent matched isotype controls. (C) Geometric mean fluorescence intensity (MFI) in NGF-depleted cells infected with 5 MOI of rgRSV was compared with the scrambled siRNA control. Values were subtracted from the isotype MFI and presented as mean±SEM (n = 4 experiments). ** = *p*<0.01 compared to controls transfected with scrambled siRNA.

On the basis of these results, we assessed whether the depletion of NGF induced by RNA interference, and the consequent overexpression of p75^NTR^ receptors, trigger apoptosis in RSV-infected bronchial epithelial cells, which had shown the highest susceptibility to RSV infection and are the natural target of clinical RSV disease. To this end, extensive FACS analysis and immunocytochemical studies were performed, and results obtained by annexin V-PE staining (Figure 7) indicated that early markers of programmed cell death are detected in human-derived bronchial epithelial cells subsequent to rgRSV exposure and that selective NGF knock-down via siRNA enhances dramatically apoptotic death driven by rgRSV infection (p<0.01).

**Figure 8:**
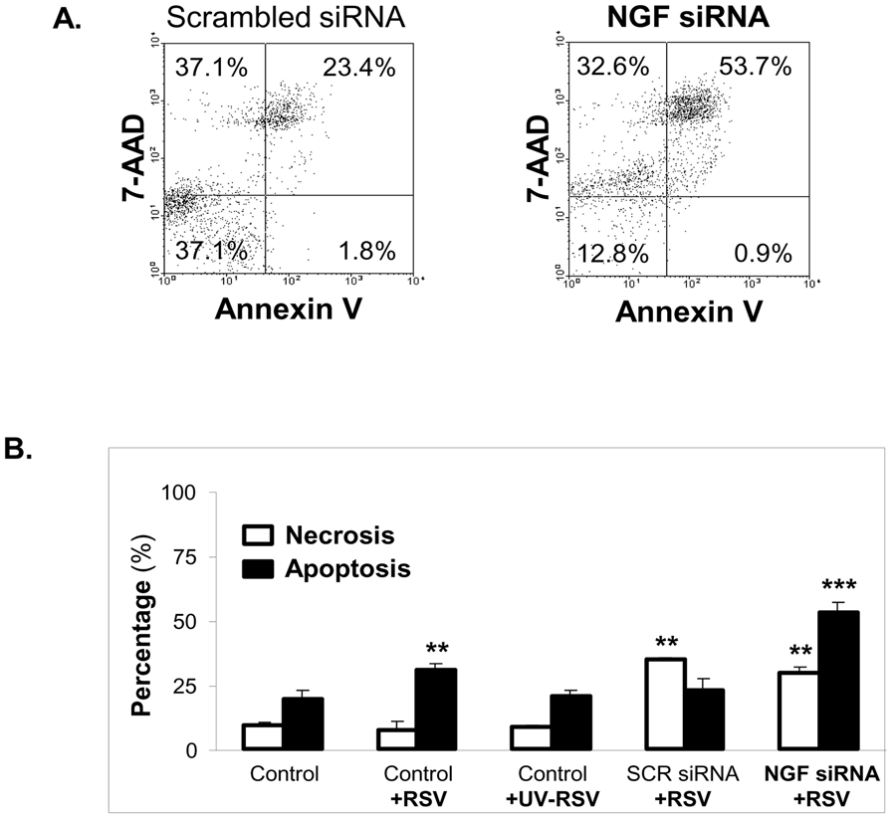
Apoptosis vs. necrosis in RSV-infected bronchial epithelial cells after NGF knock-down. (A). Dot plots of simultaneous annexin V and 7-aminoactinomycin D (7-AAD) staining of rgRSV-infected bronchial epithelial cells transfected with scrambled (SCR) siRNA (left) or with NGF-specific siRNA (right). After 48 h of transfection, cells were infected with 5 MOI of rgRSV. Apoptosis and necrosis were detected by DNA labeling with 7-AAD-PE and annexin V-Cy3 respectively. Data are expressed as percentage (%) of total cells. (B). Apoptosis vs. necrosis assessed by annexin V and 7-AAD staining followed by flow cytometry. Non-transfected controls include non-infected, rgRSV-infected, and UV-RSV-exposed cells. Data are expressed as the mean±SEM (n = 3 experiments). ** = *p*<0.01; *** = *p*<0.001 compared to non-infected controls.

We also compared the effects NGF depletion on necrosis vs. apoptosis in rgRSV-infected bronchial epithelial cells by simultaneous analysis of annexin V-Cy3 staining and 7-AAD uptake (Figure 8A). NGF-expressing cells infected with replicating rgRSV (Figure 8B) showed a small increase in apoptosis (p<0.01) and no change in necrosis compared to non-infected controls (p = 0.80), whereas UV-inactivated virus was again inert in terms of both apoptosis (p = 0.55) and necrosis (p = 0.80). NGF-depleted cells infected with rgRSV showed a significant increase in apoptosis compared to the scrambled siRNA-treated infected cells (p<0.001), whereas the change in percentage of necrotic cells was not significant (p = 0.06). All transfected cells showed more necrosis (p<0.01) compared to non-transfected controls, probably due to a non-specific toxic effect of the transfection medium (Lipofectamine 2000™).

**Figure 9:**
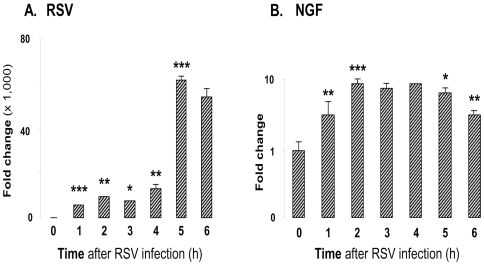
NGF transcripts induction in bronchial epithelial cells exposed to RSV. (A) RSV transcript levels. (B) NGF transcript levels. Bronchial epithelial cells were infected with 1 MOI of rgRSV. Cells were trypsinized at hourly intervals and cell pellets were collected for RNA extraction and real-time PCR analysis. Data shown are the average of 3 independent experiments performed in duplicate, and are presented as the mean±SEM (n = 6). * = *p*<0.05; ** = *p*<0.01; *** = *p*<0.001 compared to previous time point.

To substantiate further our hypothesis that NGF is an immediate response gene potentially positioned to regulate the survival of bronchial epithelial cells during RSV infection, we timed the early-phase cellular response to the infection monitoring viral and NGF transcript levels by real-time PCR at hourly intervals for the first 6 h of incubation. The data shown in Figures 9A and 9B confirm that bronchial cells respond with an immediate and persistent upregulation of NGF expression during the early phase of the RSV infection.

## Discussion

The present study suggests that among cell lines derived from the human respiratory tract, bronchial epithelial cells are especially prone to RSV infection compared to nasal and tracheal epithelial cells. This tropism may derive from the fact that NGF is strongly and rapidly upregulated in bronchial cells invaded by RSV, and may represent for these cells an essential autocrine and/or paracrine survival factor deployed early during the infection to prevent or delay apoptotic cell death caused by the virus. Furthermore, consistent with our previous studies *in vivo* [9], UV inactivation of the viral nucleic acid hindered the effect of RSV on both NGF upregulation and apoptotic cell death, indicating that the changes observed in the neurotrophin pathways are linked to active viral replication and expression of the viral genome in the respiratory epithelium. In light of this finding, it is also unlikely any contribution from non-viral factors released from the cells used for the preparation of RSV suspensions.

Our data also provide the first evidence that discrete cellular responses to RSV occur in different sections of the respiratory tract during the infection resulting in sharply different patterns of neurotrophin expression, and that in this context the expression of other neurotrophic factors and receptors is affected by NGF expression. These observations provide a critical framework for the interpretation of *in vivo* animal and clinical data derived from pooled samples obtained by airway lavage or tissue homogenization.

### RSV infection

Of initial interest in this investigation was to determine how individual cells respond to RSV infection within different anatomical regions of the respiratory tract, and their relative susceptibility to RSV. We selected the cell lines utilized in this study based on the fact that RSV makes a substantial contribution to both upper and lower respiratory tract disease in individuals of all ages [23], [24]. However, clinical evidence and *in vivo* animal models support the notion that the bronchial epithelium and associated respiratory tract is the prime target of RSV infection [25], [26]. Moreover, the epidemiological evidence from retrospective and prospective studies supports the association between early-life RSV lower respiratory tract illness and persistent airway inflammation and hyperreactivity.

Inoculation of the nose or eyes occurs by large particle aerosols or direct contact, results in viral replication in the nasopharynx with an incubation period of 4 to 5 days, and can be followed over the next several days by spread to the lower respiratory tract [27], [28] associated with the induction and secretion of multiple cytokines and chemokines [15]. In accordance with these observations, we performed our experiments with cells from distinct airway sections to reenact the initiation (nasal cells), spreading (tracheal cells), and final stabilization (bronchial cells) of the infection. These *in vitro* studies suggest that human nasal, tracheal and bronchial epithelial cells are all susceptible to RSV, but the infectivity increases moving towards the periphery following a gradient that fits well the pathological and clinical progression of RSV disease [29].

### Neurotrophin expression

NGF is the prototypical member of a family of neurotrophic factors [30], including also BDNF and the neurotrophins 3 and 4 (NT3 and NT4), which controls multiple aspects of neuronal cells life, like survival, proliferation, differentiation, neurite growth, and neurotransmission [11], [12], [31]. Two types of membrane receptors mediate neurotrophic signaling: a) the three members of the tropomyosin-related kinase (trk) family of receptor tyrosine kinases trkA, trkB and trkC, which exhibit selectivity for NGF, BDNF/NT4, and NT3 respectively; and b) the p75^NTR^ receptor, which is a member of the tumor necrosis factor (TNF) receptor superfamily [32] and binds at low affinity all four neurotrophins but can also bind at high affinity the NGF precursor (pro-NGF) and regulate the affinity of trkA for its cognate ligand. In addition to its neurotrophic functions, NGF modulates the activity of multiple non-neural cells involved in immune and inflammatory responses [33], thus functioning as a powerful and eclectic neuro-immunomodulator.

RSV infection or uptake by respiratory epithelial cells results in widespread changes in cellular gene expression involving a variety of factors, including cytokines, chemokines, cell surface molecules, and surfactants [34]. In particular, we have shown that RSV infection in rats increases the expression of NGF and its receptors in lung tissues homogenates, an effect that is exquisitely age-dependent and substantially greater in young versus older animals [9]. Overexpression of these neurogenic factors during critical developmental windows might potentially have long-term consequences distinct from adult exposures. Similar increases in NGF, its high-affinity receptor trkA, and the related neurotrophin BDNF have also been found in cells recovered by lavage from the lower airways of infants with severe RSV disease and compared to controls without respiratory infections or infected with other (parainfluenza, adenovirus) respiratory viruses [10].

In the present study, epithelial cells proximal to the bronchial tree, both nasal and tracheal, did not modify their NGF expression after being infected by RSV, and this implies that the previously reported human data obtained by bronchoalveolar lavage [10] reflect the activity of the distal airway epithelium. In contrast, we were not able to identify a clear source for the increased concentration of BDNF in RSV-infected human airways [10], suggesting that this neurotrophin is primarily contributed by infiltrating inflammatory cells. Similarly, the increase in p75^NTR^ mRNA transcripts measured in RSV-infected rat lungs [9] must have an inflammatory origin because infected airway cells actually reduce the expression of this receptor. Collectively, these data suggest that NGF may be useful as a specific biomarker of lower respiratory tract involvement during RSV infection, and also explain why several previous attempts to detect NGF in nasopharyngeal samples from RSV-infected children have failed (G. Piedimonte, unpublished observations).

Nasal and tracheal epithelial cells were more resistant to RSV infection and had a higher expression of trkA receptor, but they did not show any significant upregulation of NGF and only moderate changes in BDNF and its receptors. In contrast, bronchial epithelial cells were more susceptible to RSV infection and also showed a significant NGF upregulation subsequent to infection, suggesting that the availability of NGF may affect the efficiency of RSV infection. NGF has been shown to be involved in cell protection within the nervous and immune systems during viral infections, and may confer resistance against other virus [35]. Thus, the possibility exists that overexpression of these neurotrophic factors in RSV infected cells might serve as an innate protective mechanism, either alone or synergistically with other neurokines and cytokines [35], which allows them to tolerate, and maybe even exploit, potentially pathogenetic virus.

On the other hand, NGF represents an essential link between RSV-infected epithelial cells and the dense sub-epithelial neural networks and exerts multiple actions on immune and inflammatory cells, thereby contributing to the development and amplification of airway inflammation and hyperreactivity during and after the infection [36]. It is possible that, in addition to the absolute expression of individual neurotrophic factors and receptors, the relative expression of these molecules may also have important pathophysiologic significance for airway inflammation and remodeling.

### NGF and apoptosis

NGF is known to prevent apoptosis, or programmed cell death, of rat peritoneal mast cells through trkA [29]. TrkA stimulation also protects monocytes by up-regulating the expression of the anti-apoptotic Bcl-2 family members [37]. The trophic effects of NGF depend on the relative levels of trkA and p75^NTR^ receptors and their combined ability to form high-affinity sites on target cells. On the other hand, p75^NTR^ signaling increases JNK, NF-κB and ceramide expression and serves as a pro-apoptotic receptor.

In our study, analysis of the early-phase of RSV infection in airway epithelial cells demonstrates that infected cells actively produce NGF, express enhanced levels of trkA, and drastically decrease p75^NTR^. These adjustments protect cells from the apoptotic effects of RSV infection without affecting necrosis, thereby enhancing epithelial viability with a mechanism similar to that previously described in macrophages infected with human immunodeficiency virus (HIV) [38]. In fact, the survival of bronchial epithelial cells was dramatically decreased when the endogenous NGF supply was depleted by transfection with specific siRNA prior to RSV infection. The transcripts for p75^NTR^ increased remarkably in bronchial cells infected with RSV following NGF knock-down, pointing to the fact that p75^NTR^ can act as a major mediator of the signaling pathway responsible for apoptosis of the bronchial cells [39], [40], [41].

The relevance of these findings is closely related to the role of apoptosis as a host cell mechanism which, in contrast to necrosis, limits viral replication, propagation, and inflammation [42], [43]. Therefore, it is conceivable that the anti-apoptotic activity of NGF may, especially in conditions of relative immunological weakness, allow the persistence of low-grade RSV infection, thus providing a plausible pathophysiologic mechanism for post-bronchiolitis asthma in children and COPD in elderly adults. This hypothesis may also have important translational implications, as the selective molecular targeting of NGF during latent infections would become critical for the management of the chronic sequelae of RSV infection by improving the efficiency of viral clearance.

In conclusion, our data suggest that RSV infection of the distal (bronchial) airway epithelium, but not of the more proximal (nasal and tracheal) sections, results in overexpression of NGF and its trkA receptor, while the other p75^NTR^ receptor is markedly downregulated. This pattern of neurotrophin expression confers protection against virus-induced apoptosis, and its inhibition via RNA interference amplifies programmed cell death in the infected bronchial epithelium. The different patterns of neurotrophin expression observed between cells of proximal and distal origin also imply that the biological mechanisms of RSV infection need to be carefully considered across anatomically distinct regions, as the cellular responses to this virus are significantly different. Pharmacologic modulation of neurotrophic factors or receptors, for example using novel strategies based on RNA interference methodology, may offer a promising new approach for the management of this common respiratory infection, although additional basic and clinical research work is needed.
